# Eleven-Year Diagnostic Delay in Episodic Cluster Headache With a Characteristic International Classification of Headache Disorders, 3rd Edition (ICHD-3) Clinical Pattern: A Case of Migraine Misdiagnosis and Otologic Confusion

**DOI:** 10.7759/cureus.114845

**Published:** 2026-08-20

**Authors:** Seungyoung Kang, Amal Adi, Padma V Shetty, Asma Alskaf

**Affiliations:** 1 Faculty of Medicine, Bogomolets National Medical University, Kyiv, UKR; 2 Department of Medicine, Thumbay University Hospital, Ajman, ARE; 3 Department of Emergency Medicine, Thumbay University Hospital, Ajman, ARE; 4 Department of Neurology, Thumbay University Hospital, Ajman, ARE

**Keywords:** cluster headache, diagnostic delay, episodic cluster headache, ichd-3, migraine misdiagnosis, migraine without aura, otogenic otalgia, otologic, trigeminal autonomic cephalalgia, unilateral headache

## Abstract

Cluster headache is a primary headache disorder characterized by recurrent attacks of severe unilateral headache accompanied by ipsilateral cranial autonomic symptoms. Despite well-established diagnostic criteria, it remains one of the most frequently misdiagnosed primary headache disorders, resulting in substantial diagnostic delay. We report a diagnostic dilemma involving a 31-year-old man with episodic cluster headache who remained undiagnosed for 11 years despite a clinical presentation highly consistent with the International Classification of Headache Disorders, 3rd edition (ICHD-3) criteria. Throughout the disease course, he experienced recurrent severe unilateral orbital pain lasting 30-60 minutes, occurring more than three times daily during annual cluster periods and accompanied by ipsilateral lacrimation, nasal congestion, rhinorrhea, and marked restlessness. However, he was repeatedly diagnosed with migraine without aura and later underwent ophthalmologic and otolaryngologic evaluations, further delaying recognition of the characteristic headache pattern. Detailed reassessment of the clinical history ultimately led to a clinical diagnosis of episodic cluster headache, and guideline-recommended therapy was followed by substantial clinical improvement. The principal educational feature of this case is how prominent ipsilateral ear pain and distracting otologic and ophthalmologic findings contributed to delayed recognition of an otherwise characteristic cluster headache presentation. Careful assessment of attack duration, periodicity, cranial autonomic symptoms, and restlessness remains essential for timely recognition of cluster headache, particularly when apparently relevant local findings do not adequately explain the overall headache pattern.

## Introduction

Cluster headache is a primary headache disorder belonging to the trigeminal autonomic cephalalgias (TACs) and is characterized by recurrent attacks of severe unilateral orbital, supraorbital, or temporal pain lasting 15-180 minutes, accompanied by ipsilateral cranial autonomic symptoms and/or marked restlessness or agitation, according to the International Classification of Headache Disorders, 3rd edition (ICHD-3) [1]. Although its clinical phenotype is distinctive, cluster headache remains one of the most frequently misdiagnosed primary headache disorders, often resulting in years of unnecessary suffering and inappropriate treatment [2-4].

Recent evidence indicates that the average diagnostic delay for cluster headache remains approximately 10 years despite increasing clinical awareness [5,6]. Previous studies have consistently shown that migraine is the most common initial misdiagnosis, with many patients undergoing repeated healthcare consultations before the correct diagnosis is established [2,3,5-7]. Prolonged diagnostic delay has been associated with ineffective treatment, repeated investigations, increased healthcare utilization, impaired quality of life, and substantial psychological burden [3,5,6]. Although migraine is the most common initial misdiagnosis, careful assessment of attack duration, circadian and circannual periodicity, ipsilateral cranial autonomic symptoms, and restlessness readily distinguishes cluster headache from migraine [1,2,4]. In addition, referred otalgia caused by shared trigeminal and other cranial sensory pathways may further obscure the diagnosis and lead to unnecessary otolaryngologic evaluation [4,8,9].

We present a diagnostic dilemma involving a patient with episodic cluster headache who remained undiagnosed for 11 years despite an otherwise characteristic clinical presentation. Although prolonged diagnostic delay and migraine misdiagnosis are well documented in cluster headache, the principal educational feature of this case is the prominent ipsilateral ear pain that prompted otolaryngologic evaluation, together with unrelated ophthalmologic findings that further distracted from recognition of the underlying headache disorder. This case emphasizes the importance of reassessing the overall headache pattern when apparently relevant local findings do not adequately explain the patient's recurrent symptoms.

## Case presentation

A 31-year-old man presented with an 11-year history of recurrent severe unilateral headaches occurring approximately once annually. Each active headache period lasted approximately two to three months and was followed by prolonged symptom-free remission.

Individual attacks consisted of excruciating stabbing pain centered in the right orbital region with radiation to the ipsilateral ear and occipital region. The attacks lasted 30-60 minutes, occurred more than three times daily during active periods, and predominantly occurred at night. The patient described the pain as “suicidal” because of its extreme intensity. Headache attacks were consistently accompanied by ipsilateral lacrimation, nasal symptoms, and marked restlessness, often resulting in pacing and emotional distress. The patient denied nausea and vomiting, and no prominent migrainous features were identified in the detailed history. Despite this stereotyped presentation, the patient sought medical attention at multiple healthcare institutions over an 11-year period and was repeatedly diagnosed with migraine without aura. Multiple anti-migraine medications were prescribed without meaningful symptom relief.

Approximately seven months before the definitive diagnosis, the patient underwent ophthalmologic evaluation for visual discomfort and was diagnosed with bilateral superficial keratitis, acute conjunctivitis, and visual deprivation nystagmus. These bilateral ocular conditions were not temporally associated with the unilateral headache attacks and were considered unrelated to the underlying headache disorder. Approximately four months before diagnosis, the patient underwent otolaryngologic evaluation for ipsilateral ear pain and was diagnosed with otogenic otalgia. Otolaryngologic examination revealed impacted cerumen without evidence of significant external or middle ear disease. Removal of the impacted cerumen did not alter the patient's characteristic headache pattern.

**Figure 1:**
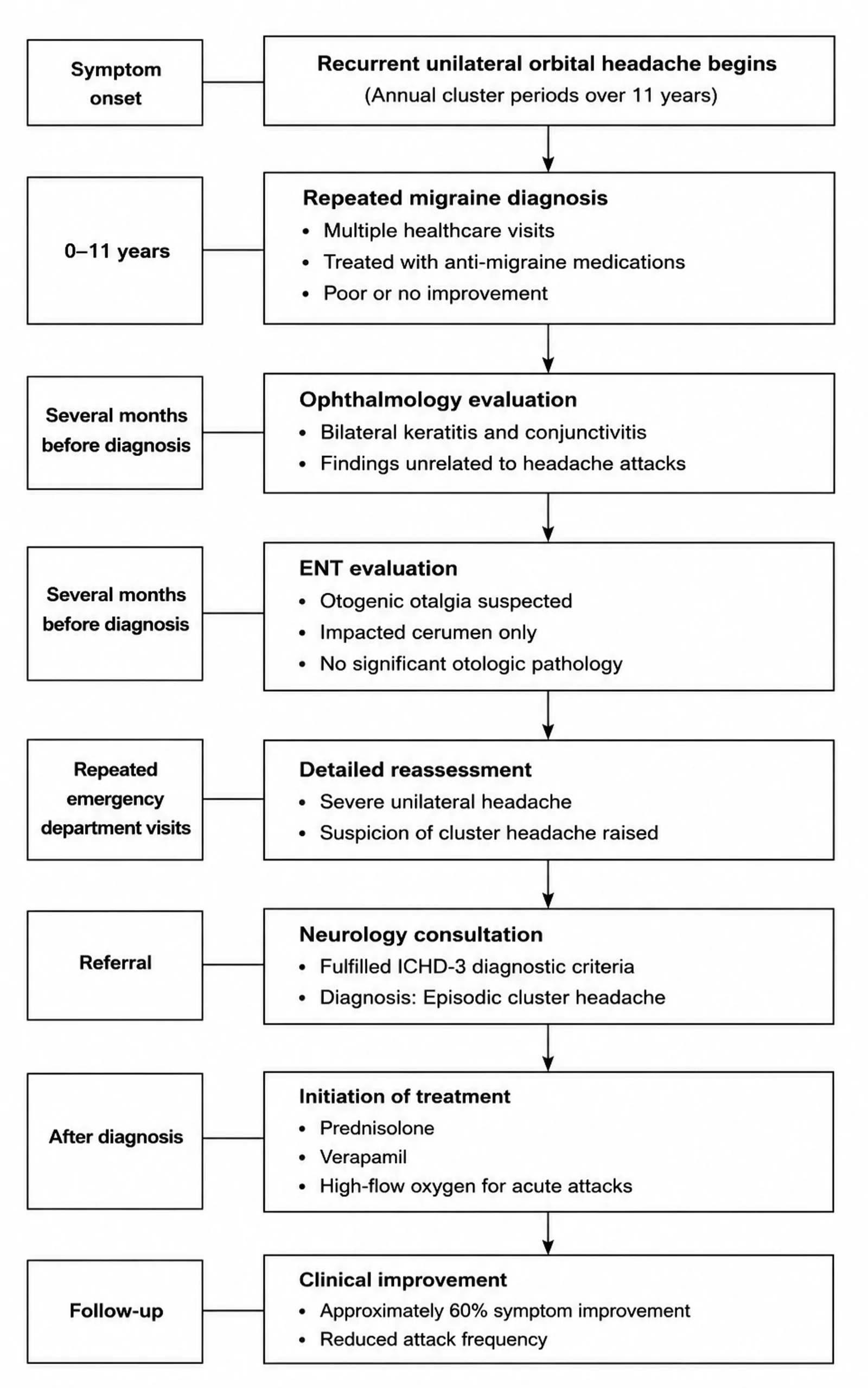
Diagnostic journey from symptom onset to the diagnosis of episodic cluster headache. This figure illustrates the chronological clinical course from symptom onset to the final diagnosis. Despite recurrent stereotyped attacks characterized by severe unilateral orbital pain, cranial autonomic symptoms, and annual cluster periods, the patient was repeatedly diagnosed with migraine without aura. Subsequent ophthalmologic and otolaryngologic evaluations further contributed to the diagnostic delay, until recognition of the characteristic headache pattern prompted neurological assessment and the diagnosis of episodic cluster headache. ICHD-3: International Classification of Headache Disorders, 3rd edition.

The patient subsequently presented to our emergency department with severe unilateral headache and was initially managed as migraine. During several repeat visits over the following days, a more detailed headache history revealed a stereotyped attack pattern of short-lasting unilateral orbital pain associated with ipsilateral cranial autonomic symptoms, annual periodicity, and marked restlessness. These findings prompted reconsideration of the initial diagnosis and raised strong clinical suspicion for episodic cluster headache. The patient's diagnostic journey is summarized in Figure 1.

**Figure 2:**
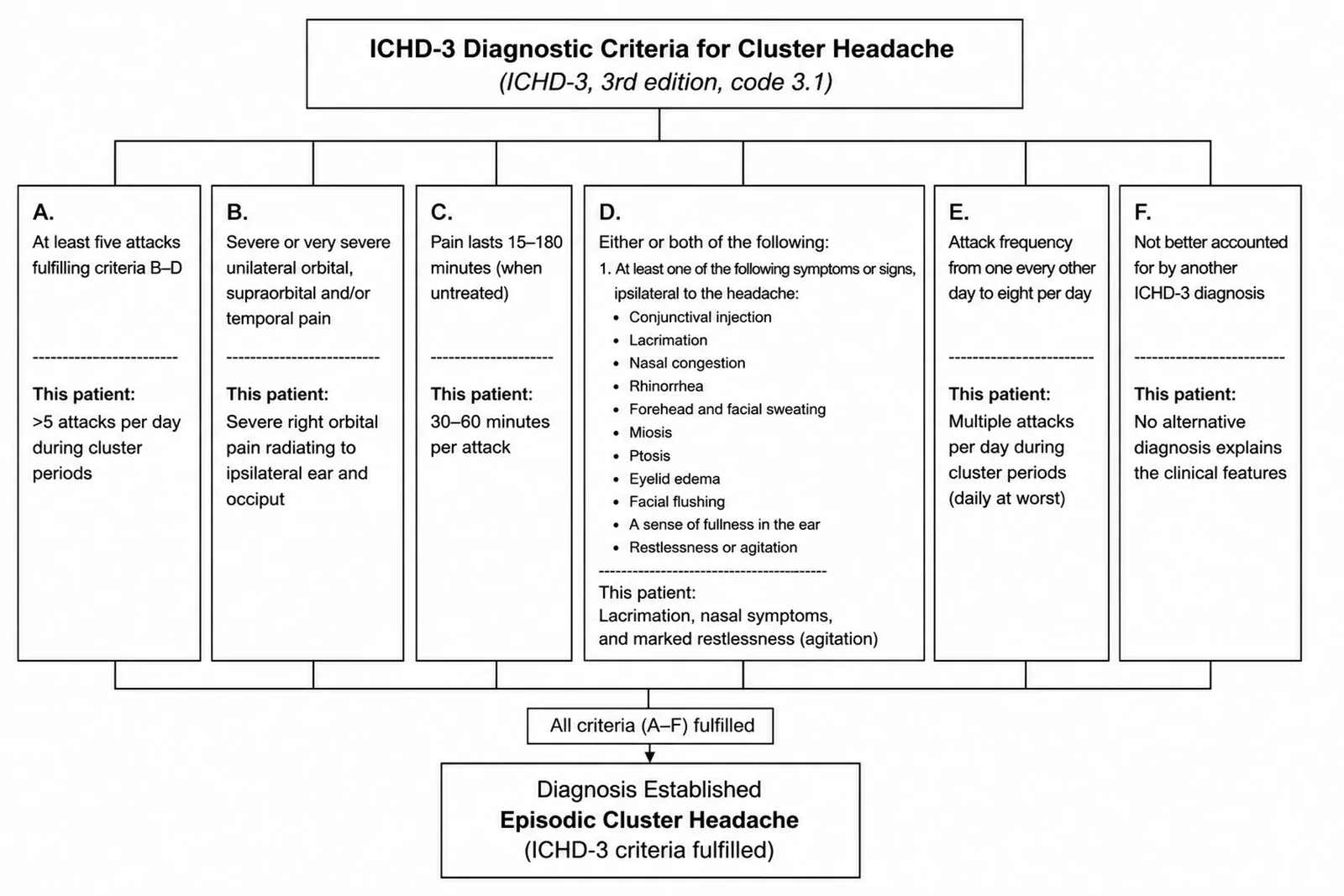
Application of the International Classification of Headache Disorders, 3rd edition (ICHD-3) diagnostic criteria in the present case. This figure demonstrates the correspondence between the patient's clinical features and each ICHD-3 diagnostic criterion for episodic cluster headache. The patient's clinical features were highly consistent with the ICHD-3 diagnostic criteria for episodic cluster headache; however, secondary causes of cluster-like headache could not be completely excluded because neuroimaging was not performed. ICHD-3: International Classification of Headache Disorders, 3rd edition.

Neurological consultation established the clinical diagnosis of episodic cluster headache based on the patient's history and headache phenotype, which was clinically consistent with the ICHD-3 diagnostic criteria (Figure 2) [1].

No previous neurological consultation had been documented despite multiple healthcare visits over the preceding 11 years. Neuroimaging was recommended to exclude secondary causes but was deferred because of financial constraints [4,10]. Given the characteristic clinical history and absence of red-flag features, treatment was initiated with oral prednisolone as transitional therapy and verapamil for preventive management [11].

Before treatment, attacks occurred daily, predominantly during the night. Following initiation of oral prednisolone and verapamil, the patient reported a reduction in headache frequency from daily attacks to approximately one attack every three days and subjectively estimated an overall improvement of approximately 60%. During an acute attack following the diagnosis of cluster headache, high-flow oxygen at 12-15 L/min resulted in complete resolution of symptoms within 30 minutes [11,12]. During approximately four months of follow-up, no further headache-related hospital visits were documented in the available medical records, although the patient continued regular outpatient follow-up for medication management. No standardized headache outcome measure was used.

## Discussion

The principal educational feature of this case is how prominent ipsilateral ear pain and distracting otologic and ophthalmologic findings contributed to delayed recognition of an otherwise characteristic presentation of episodic cluster headache. Although the 11-year diagnostic delay and repeated migraine misdiagnosis are consistent with previously reported experience in cluster headache [2,5-7], the combination of ear-directed pain, limited otologic findings, and unrelated ophthalmologic abnormalities complicated recognition of the underlying headache disorder in this patient. The recurrent stereotyped headache pattern was highly consistent with the clinical features described in the ICHD-3 criteria [1]. Recent evidence has demonstrated generally high concordance between clinical headache diagnoses and ICHD-3 criteria when these criteria are systematically applied, emphasizing the importance of structured clinical assessment [13]. In addition to diagnostic anchoring on migraine, availability bias may have contributed because migraine is substantially more common and may therefore be more readily considered in patients presenting with recurrent unilateral headache. Premature closure may have further reinforced the delay when findings such as impacted cerumen or unrelated ophthalmologic abnormalities were accepted as sufficient explanations before the overall headache pattern was reassessed [4].

Delayed diagnosis remains a well-recognized challenge in cluster headache [2,3,8]. A recent systematic review and meta-analysis reported a mean diagnostic delay of approximately 10.4 years, indicating that prolonged delays remain common despite increased awareness [5]. Misdiagnosis is also common. In a hospital-based study of 144 patients with episodic cluster headache, 77% were misdiagnosed at the first medical consultation; the most frequent alternative diagnoses were trigeminal neuralgia (22%), migraine without aura (19%), and sinusitis (15%) [7]. A systematic review including 4,661 patients likewise found that diagnostic delay, multiple alternative diagnoses, and consultations with several different clinicians were widespread across healthcare systems [6]. Our patient's 11-year delay is therefore consistent with published literature rather than an isolated occurrence.

Migraine without aura was the principal alternative diagnosis throughout this patient's clinical course. Although migraine and cluster headache may both present with recurrent unilateral headache, several clinical features clearly favored cluster headache in this case [1-3]. The distinguishing clinical features between episodic cluster headache and migraine are summarized in Table 1.

**Table 1:** Comparison of the clinical characteristics of episodic cluster headache and migraine, highlighting the features observed in the present case. This table summarizes the key clinical differences between episodic cluster headache and migraine and demonstrates how the patient's clinical presentation was more consistent with episodic cluster headache, facilitating the final diagnosis. Information synthesized from the ICHD-3 diagnostic criteria and published literature [1,2,13]. CGRP: calcitonin gene-related peptide; NSAID: nonsteroidal anti-inflammatory drug.

Clinical feature	Episodic cluster headache	Migraine	Findings in the present case
Pain location	Strictly unilateral orbital, supraorbital, or temporal pain	Usually unilateral but may alternate or become bilateral	Strictly right orbital pain radiating to the ipsilateral ear and occiput
Pain severity	Severe to very severe; often described as excruciating	Moderate to severe	Excruciating, described as “suicidal” pain
Attack duration	15-180 minutes	4-72 hours	30-60 minutes
Attack pattern	Recurrent attacks during cluster periods with prolonged remission	Episodic attacks without characteristic cluster periodicity	Annual cluster periods lasting weeks to months
Cranial autonomic symptoms	Common (lacrimation, nasal congestion/rhinorrhea, conjunctival injection, etc.)	Uncommon or mild	Ipsilateral lacrimation, nasal congestion and rhinorrhea
Patient behavior during attacks	Restlessness or agitation; inability to remain still	Preference for lying still in a dark, quiet room	Marked agitation, pacing, and emotional distress
Response to acute therapy	High-flow oxygen and subcutaneous/intranasal triptans	NSAIDs, triptans, antiemetics	Rapid relief with high-flow oxygen
Preventive therapy	Verapamil is first-line preventive therapy	Preventive therapy varies (e.g., beta-blockers, topiramate, CGRP-targeted therapies)	Improved after prednisolone transition and verapamil
Key diagnostic clue	Short-lasting unilateral attacks with cranial autonomic symptoms and restlessness	Longer attacks, commonly associated with nausea, photophobia, and preference for rest	Short-lasting unilateral attacks with ipsilateral cranial autonomic manifestations and marked restlessness

The attacks were short (30-60 minutes), occurred in circannual cluster periods, were accompanied by ipsilateral lacrimation and nasal symptoms, and caused marked agitation. These features differ from the longer attack duration and preference for rest that are more typical of migraine [1,2]. This case illustrates how diagnostic anchoring on a common disorder may delay recognition of a less prevalent but clinically distinctive condition.

Otologic symptoms represented an important source of diagnostic distraction in this case. The patient underwent otolaryngologic evaluation for ipsilateral ear pain, although examination revealed no significant pathology other than impacted cerumen, and cerumen removal did not alter the recurrent headache pattern. The sensory innervation of the ear is complex and involves multiple cranial and upper cervical nerves [8,9]. Although a relationship between the ear pain and the underlying headache disorder is clinically plausible, its mechanism cannot be established from this case. The persistence of severe unilateral ear pain despite limited otologic findings should therefore prompt consideration of non-otologic sources and careful assessment of the accompanying headache phenotype.

The patient also underwent ophthalmologic evaluation before the diagnosis of cluster headache. Although bilateral superficial keratitis and conjunctivitis were identified, these conditions were temporally unrelated to the unilateral headache attacks and did not explain the recurrent unilateral headache pattern. Their presence nevertheless contributed to diagnostic distraction, emphasizing the importance of distinguishing coexisting ocular disease from the unilateral cranial autonomic manifestations typical of cluster headache.

Cluster headache is primarily diagnosed clinically through careful history-taking and assessment of the characteristic attack pattern according to the ICHD-3 criteria [1,4,10]. Neuroimaging was recommended to evaluate for potential secondary causes but was not performed because of financial constraints. Consequently, although the clinical presentation was highly consistent with episodic cluster headache, secondary causes of cluster-like headache could not be completely excluded, which represents an important limitation of this case.

The patient's stereotyped attack pattern and fulfillment of the ICHD-3 diagnostic criteria provided the primary basis for the clinical diagnosis. Although neuroimaging was not performed, the clinical history was highly consistent with episodic cluster headache, and the favorable response to guideline-recommended therapy was considered supportive rather than diagnostic [1,10-12]. The marked improvement following treatment with prednisolone 60 mg daily and verapamil 80 mg twice daily, together with the rapid response to high-flow oxygen (12-15 L/min), was consistent with current treatment recommendations for episodic cluster headache [10-12].

Overall, this case emphasizes the importance of reassessing an initial working diagnosis when the duration, periodicity, cranial autonomic manifestations, and patient behavior during attacks point toward a different and clinically recognizable headache syndrome [1,5]. Apparently relevant otologic or ophthalmologic findings should not preclude reassessment of the broader headache pattern when they do not adequately explain the patient's recurrent symptoms.

## Conclusions

This case highlights how an otherwise characteristic presentation of episodic cluster headache may remain unrecognized for years despite being highly consistent with the clinical features described in the ICHD-3 criteria. In this patient, prominent ipsilateral ear pain and unrelated ophthalmologic abnormalities contributed to diagnostic distraction during a prolonged clinical course. Careful assessment of attack duration, periodicity, unilateral cranial autonomic manifestations, and restlessness remains essential for timely recognition and appropriate neurological referral, particularly when otologic or ocular findings do not adequately explain the overall headache pattern.
